# Supplementary material for: A high-resolution mRNA expression time course of embryonic development in zebrafish
Source: eLife. 2017 Nov 16;6:e30860. doi: 10.7554/eLife.30860 (PMC5690287; doi:10.7554/eLife.30860)
Supplement: Supplementary file 6. [file elife-30860-supp6.zip › biolayout-clusters-files/Cluster091-genes.html]

Cluster091


# Cluster091: Genes

| Ensembl ID | Gene Name | Chr | Start | End | Biotype |
| --- | --- | --- | --- | --- | --- |
| ENSDARG00000094730 | acbd7 | 16 | 28661932 | 28664705 | protein\_coding |
| ENSDARG00000075444 | cgref1 | 17 | 32678278 | 32684022 | protein\_coding |
| ENSDARG00000055009 | col4a1 | 9 | 8652642 | 8691828 | protein\_coding |
| ENSDARG00000079280 | dhrsx | 1 | 32651142 | 32753395 | protein\_coding |
| ENSDARG00000075021 | fam120a | 11 | 27457856 | 27491875 | protein\_coding |
| ENSDARG00000045608 | fam19a5b | 25 | 29470091 | 29545041 | protein\_coding |
| ENSDARG00000036156 | fnbp1a | 8 | 11537179 | 11602610 | protein\_coding |
| ENSDARG00000099732 | gfra1a | 13 | 20261674 | 20388019 | protein\_coding |
| ENSDARG00000018228 | htr2cl1 | 7 | 23086740 | 23223452 | protein\_coding |
| ENSDARG00000021480 | olfml2bb | 2 | 20946803 | 20957662 | protein\_coding |
| ENSDARG00000011122 | pptc7a | 8 | 4311828 | 4345475 | protein\_coding |
| ENSDARG00000075245 | wdr11 | 13 | 2599773 | 2695040 | protein\_coding |
| ENSDARG00000078034 | zgc:112001 | 20 | 99533 | 101620 | protein\_coding |
| ENSDARG00000069549 | zgc:162396 | 9 | 28877828 | 28881226 | protein\_coding |
